# Seabed Modelling by Means of Airborne Laser Bathymetry Data and Imbalanced Learning for Offshore Mapping

**DOI:** 10.3390/s22093121

**Published:** 2022-04-19

**Authors:** Tomasz Kogut, Arkadiusz Tomczak, Adam Słowik, Tomasz Oberski

**Affiliations:** 1 Department of Geodesy and Offshore Survey, Maritime University of Szczecin, Żołnierska 46, 71-250 Szczecin, Poland; a.tomczak@am.szczecin.pl; 2 Department of Computer Engineering, Koszalin University of Technology, Sniadeckich 2, 75-453 Koszalin, Poland; adam.slowik@tu.koszalin.pl; 3 Department of Geodesy and Geoinformatics, Koszalin University of Technology, Sniadeckich 2, 75-453 Koszalin, Poland; tomasz.oberski@tu.koszalin.pl

**Keywords:** airborne laser bathymetry, imbalanced learning, classification, SMOTE, oversampling

## Abstract

An important problem associated with the aerial mapping of the seabed is the precise classification of point clouds characterizing the water surface, bottom, and bottom objects. This study aimed to improve the accuracy of classification by addressing the asymmetric amount of data representing these three groups. A total of 53 Synthetic Minority Oversampling Technique (SMOTE) algorithms were adjusted and evaluated to balance the amount of data. The prepared data set was used to train the Multi-Layer Perceptron (MLP) neural network used for classifying the point cloud. Data balancing contributed to significantly increasing the accuracy of classification. The best overall classification accuracy achieved varied from 95.8% to 97.0%, depending on the oversampling algorithm used, and was significantly better than the classification accuracy obtained for unbalanced data and data with downsampling (89.6% and 93.5%, respectively). Some of the algorithms allow for 10% increased detection of points on the objects compared to unbalanced data or data with simple downsampling. The results suggest that the use of selected oversampling algorithms can aid in improving the point cloud classification and making the airborne laser bathymetry technique more appropriate for seabed mapping.

## 1. Introduction

Information on water depth and seabed topography can contribute to improving the safety of maritime transport and to the development of other maritime industries, including the offshore sector. Hydrographic surveying is done systematically all over the world to prepare data for nautical charts, electronic navigation systems, and other databases used in the management of hydrospace and maritime infrastructure. The airborne laser bathymetry (ALB) technique can be a valuable addition to Multibeam Echosounders (MBES) or perhaps an alternative in shallow waters. It has proven to be a large-scale, accurate, rapid, safe, and versatile approach for surveying shallow waters and coastlines where sonar systems are ineffective or impossible to use [1,2,3,4]. Research has shown that ALB can identify similar seafloor features such as MBES systems [5]. However, additional improvements must be done to separate the LiDAR seafloor intensity data from the depth component of the signal waveform. Receiving bathymetric lidar data with unassigned point classes or inaccurate point classification that may not meet industrial or research requirements is not unusual [6]. Studies that have used ALB for depth determination and object detection primarily point to challenges in classifying the resulting point cloud into three basic groups: bottom, water surface, and bottom objects. These issues can be overcome using well-recognized machine learning classification methods.

The main goal of this study is to increase the accuracy of the classification of point clouds measured by an ALB scanner to improve seabed modeling and object detection.

This paper can be considered a novel input to the ALB classification of point clouds with the use of imbalanced learning. To achieve the goal, the study evaluated Multi-Layer Perceptron (MLP) Artificial Neural Network (ANN) with the softmax activation function employing over 50 variants of the oversampling techniques for imbalanced learning. The results confirmed that data balancing had a quantitative impact on classification accuracy, allowing enhanced detection of seabed and bottom based on the ALB data. The classification results indicated that the best overall classification accuracy achieved varied from 95.8% to 97.0% depending on the oversampling algorithm used and was significantly better than the classification accuracy obtained for unbalanced data and data with downsampling (89.6% and 93.5%, respectively). Some of the algorithms allow for 10% increased detection of points on the objects compared to unbalanced data or data with simple downsampling. This study did not develop a new data balancing method or enhance the existing ones.

The classification accuracy of point clouds of all the classes is influenced by class distribution. According to the scanned area in the majority, the laser scanning data are unbalanced, and therefore require remodeling. The ALB data set of shallow waters comprises data on the seabed and a small percentage of data on underrepresented seabed objects. This application necessitates a high rate of accurate detection in the minority class (seabed objects) and a low rate of mistakes in the majority class (seabed or water surface). Different oversampling methods have been analyzed to address this concern [7]. Archaeologists focusing on detecting former field systems from LiDAR data in their research recommend the use of the Synthetic Minority Oversampling Technique (SMOTE) for achieving better results [8]. Balancing the training data for automatic mapping of high-voltage power lines based on the LiDAR data led to an almost 10% increase in accuracy in comparison to imbalanced data [9]. Landslide prediction research based on a set of geomorphological factors revealed that the Support Vector Machine (SVM) model yielded the highest accuracy with the SMOTE data balancing method [10]. The supporting synthetic samples were used in the classification of bottom materials (sand, stones, rocks) performed using ALB. The obtained results were promising but were specific for particular classes [11]. A study on the application of SMOTE for balancing data distribution with land cover mapping using LiDAR data showed increased detection accuracy. The challenges associated with imbalanced classes and low density of LiDAR point clouds in urban areas were also satisfactorily resolved by applying several oversampling methods for the classification and extraction of roof superstructures [12]. Due to its proven advantages in classification, the present study used SMOTE, a method for producing synthetic new data from existing ones, which provided new information and variations to synthetically generated data.

The paper is organized as follows: Section 2 describes the test area and ALB data with features and architecture of ANN. Section 3 presents the results obtained with the proposed approach and a discussion. Finally, Section 4 presents our conclusions.

## 2. Materials and Methods

### 2.1. Test Area

**Figure 1:**
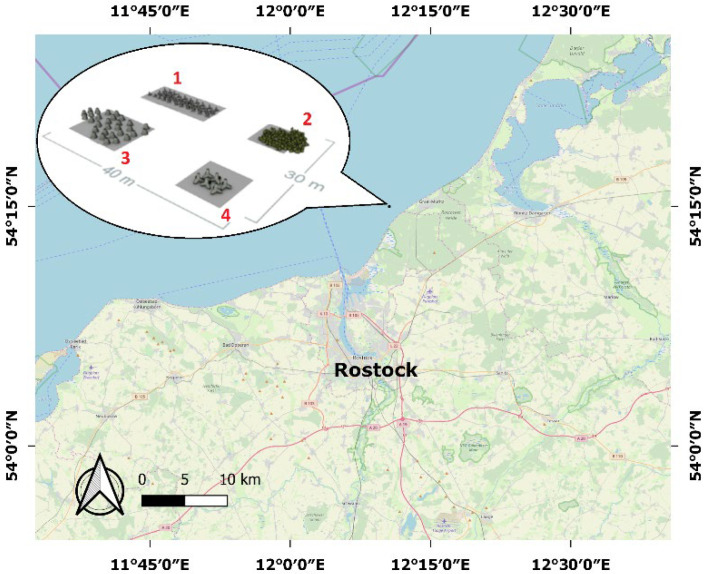
Location of the test area (approximately 25 km north of the city of Rostock in Germany).

The test area is the artificial reef Rosenort on the Baltic Sea. It is located between Markgrafenheide and Graal-Müritz (Germany), approximately 2000 m from the coast, at a water depth of 6 m. The reef is a protected fishery reserve, and thus activities such as angling, fishing, and anchoring are prohibited. The Rosenort reef is divided into four artificially constructed zones. The zones were built from (1) 52-ton concrete tetrapods, (2) 180-ton natural stones, (3) 30 cut reef cones, and (4) six 6-ton concrete tetrapods (Figure 1).

### 2.2. Point Cloud and Features

**Figure 2:**
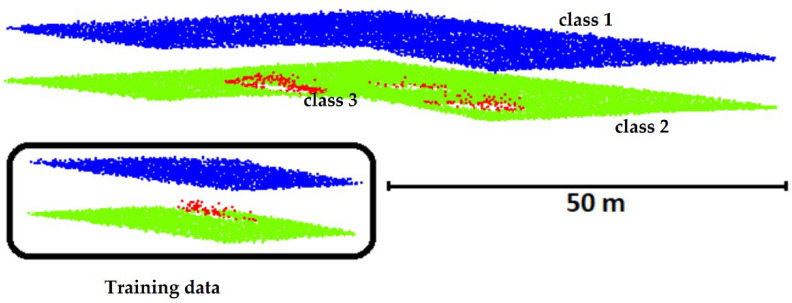
Three classes in the ALB point cloud (blue—water surface, class 1; green—seabed, class 2; red—points on the object on the seabed, class 3).

The point cloud was collected in September 2013 using an AHAB Chiroptera I scanner, at a flight altitude of 400 m. The Chiroptera I scanner is equipped with two beams and scans in an elliptical shape at an angle of 20° between the scan direction and the nadir. This laser scanner uses a near-infrared (NIR) laser with a wavelength of 1064 nm at a peak measurement frequency of 400 kHz for detecting water surfaces and a green laser with a wavelength of 532 nm at a frequency of 36 kHz for underwater measurements. The horizontal nominal accuracy of the infrared beam and the green beam is 0.2 and 0.75 m, respectively, while the depth of nominal accuracy is 0.15 m. The Secchi depth achieved with the scanner exceeded 1.5 m, and during the measurement, the depth was measured at around 6.3 m. In this study, the point cloud obtained from the green beam (Figure 2) was used for analysis. The density of the point cloud obtained for the test area was 2.6 points on the water surface and 3.3 points on the underwater point (seabed and seabed object).

**Figure 3:**
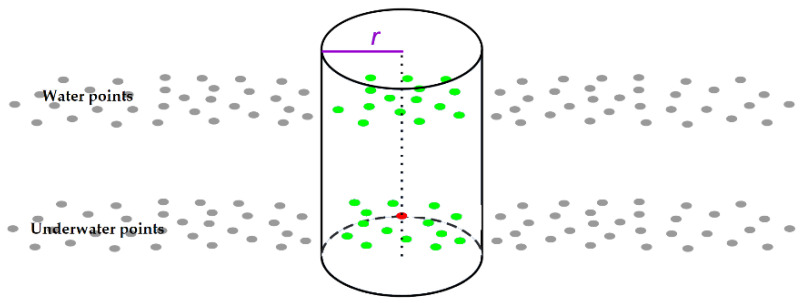
Visualization of the cylinder and analyzed points (red—analyzed point, green—points inside the cylinder used to compute the features, grey—other points in the point cloud, *r*—radius).

Scanning with the use of the AHAB Chiroptera I scanner allowed collecting the cloud of spatially coordinated points with their intensities. The analysis of such data, especially the full waveform, can provide additional information on the measured points that can aid in the classification of the acquired data. Five features (U_1_–U_5_, Table 1) derived from the full waveform, were used. A well-defined region delineated by a cylinder of a given radius r, which was 5 m (Figure 3), was used to analyze the location of each point along with its neighborhood. Features U_6_–U_15_, which describe the geometry of the point cloud, were used in the investigation.

### 2.3. Architecture of ANN

The raw (unbalanced) ALB data set used for training the ANN consisted of 6198 vectors (Figure 3, data in the black box). Each vector had 18 items describing the values of 15 input attributes (U1–U15, Table 1) and that of three output classes (U38–U40, Table 2). For the error back-propagation method, 80% of these vectors were utilized for training and 20% for validating the ANN.

The three classes were labeled as follows: class 1 (U_38_)-water surface with 2729 vectors, class 2 (U_39_)-seabed represented by 3396 vectors, and class 3 (U_40_)-seabed object containing 73 vectors. Since the classes had a different number of vectors, for training the ANN, the number of vectors in each class was balanced by applying different oversampling algorithms (Table 3, first column). The data set thus prepared, consisted of a different number of vectors (Table 3, last three columns), depending on the algorithm used. Imbalancing of data typically refers to classification tasks where the classes are not equally represented. Several approaches have been proposed for this issue. Among them, SMOTE has been widely used to produce synthetic samples between minority samples in the feature space. This technique improves class imbalance by linear interpolation between the underrepresented class samples [7]. It creates new instances of minority group data, by copying existing data and making minor changes. Moreover, SMOTE is a great tool for amplifying the already existing signal in minority groups without creating new signals for these groups. In general, synthetic samples are generated as a difference between the feature vector (sample) and its randomly chosen nearest neighbor. This difference is multiplied by a random number between 0 and 1 and added to the feature vector considered for creating a new sample —the synthetic one. Several improvements have been proposed for synthetic sample creation algorithms since the introduction of SMOTE. The present work included 53 oversampling methods, and a comparison of their results is provided in this paper. The data were standardized in a later step of data processing.

**Figure 4:**
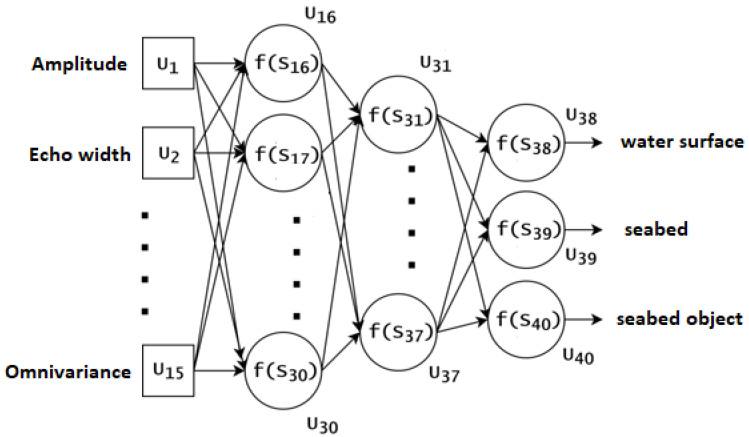
The architecture of ANN.

The ANN used in the experiments is presented in Figure 4. It is an MLP neural network [15], which has 15 inputs (U_1_–U_15_), three layers of neurons, and three outputs (U_38_–U_40_). The first layer comprises 15 neurons (U_16_–U_30_), the second layer has seven neurons (U_31_–U_37_), and the third layer has three neurons (U_38_–U_40_). Neurons in the previous layer are fully connected with those in the next layer (Figure 4). In the first layer, as well as the second layer from the bottom, all neurons possess a unipolar sigmoidal activation function, while in the last layer, all neurons (U_38_–U_40_) possess the soft-max activation function.

The values of the neural network outputs (U_38_–U_40_) inform the probability value, which indicates the degree of belonging of a given input vector to each of the three classes (water, seabed, seabed object). The ANN presented in Figure 4 was trained using an error-back propagation algorithm with the learning coefficient *ro* = 0.01. The maximal number of iterations was 1750.

## 3. Results and Discussion

The proposed approach was tested for each oversampling method by training the MLP neural network. A random starting point was used in error back-propagation. Consequently, the training procedure was repeated 11 times to obtain reliable results. After completion of each iteration, the data were tested with the dataset, which initially contained 10,612 water surface points, 13,318 seabed points, and 212 seabed object points. The results of the tests are presented in Table 3. The first two columns in the table present the names of oversampling algorithms and the year they were introduced. The next four columns show the best, worst, mean and median values of overall classification accuracy. The last four columns present the number of vectors used for training the MLP neural network.

The overall classification accuracy (*Ac* [%]) was calculated using the following formula:

(10) $$Ac=\frac{\left(\frac{cor_{w}}{all_{w}}+\frac{cor_{s}}{all_{s}}+\frac{cor_{o}}{all_{o}}\right)\times 100\%}{3}$$

where *cor_w_* is the number of input vectors successfully identified as “water surface” in class 1, *cor_s_* is the number of input vectors successfully identified as “seabed” in class 2, *cor_o_* is the number of input vectors successfully identified as “seabed object” in class 3, and *all*_{*w,s,o*}_ is the total number of vectors in classes 1–3.

The best overall classification accuracy of 97.0% was achieved for the LVQ SMOTE (Learning Vector Quantization based SMOTE) algorithm. The oversampling method generated synthetic samples using codebooks obtained by learning vector quantization [16]. The second algorithm with about 96% overall classification accuracy was ROSE (Random OverSampling Examples). This algorithm works based on smoothed bootstrap resampling from data [17]. The next algorithm with the best results was PDFOS (Probability Density Function Over-Sampling), and its overall classification accuracy was about 95.8%. This algorithm generated synthetic instances as additional training data based on the estimated probability density function [18].

**Table 3:** Results of classification with balanced learning.

	Name	Year	Best	Worst	Mean	Median	All Vectors	Class 1	Class 2	Class 3
1	SMOTE [7]	2002	93.4	91.5	92.7	92.9	10,188	3396	3396	3396
2	SMOTE + Tomek [19]	2004	93.0	91.9	92.6	92.6	10,135	3396	3396	3343
3	SMOTE + ENN [19]	2004	93.5	90.6	92.2	92.1	9990	3396	3396	3198
4	Borderline-SMOTE1 [20]	2005	93.3	91.5	92.2	92.1	9191	2729	3396	3066
5	Borderline-SMOTE2 [20]	2005	**95.4**	93.4	94.7	**94.8**	9191	2729	3396	3066
6	SMOTE + LLE [21]	2006	91.1	88.2	89.7	89.7	10,188	3396	3396	3396
7	Distance-SMOTE [22]	2007	93.5	91.9	92.5	92.5	10,188	3396	3396	3396
8	Polynomial-SMOTE [23]	2008	91.0	88.7	90.3	90.4	13,234	5458	3396	4380
9	ADOMS [24]	2008	94.2	91.4	93.3	93.5	10,188	3396	3396	3396
10	Safe Level SMOTE [25]	2009	66.7	66.7	66.7	66.7	6573	2729	3396	448
11	MSMOTE [26]	2009	94.1	92.0	92.9	92.9	10,188	3396	3396	3396
12	SMOBD [27]	2011	95.0	92.7	93.3	93.0	10,188	3396	3396	3396
13	SVM balance [28]	2012	94.2	91.9	92.7	92.5	10,172	3396	3396	3380
14	TRIM SMOTE [29]	2012	92.4	91.5	92.0	92.0	10,188	3396	3396	3396
15	SMOTE RSB [30]	2012	81.7	66.7	71.4	67.6	7716	3396	3396	924
16	ProWSyn [31]	2013	93.6	90.6	92.4	92.5	10,188	3396	3396	3396
17	SL graph SMOTE [32]	2013	92.1	91.1	91.6	91.6	9191	2729	3396	3066
18	NRSBoundary SMOTE [33]	2013	92.6	91.4	91.8	91.8	9191	2729	3396	3066
19	LVQ SMOTE [16]	2013	**97.0**	94.7	96.3	**96.7**	10,188	3396	3396	3396
20	ROSE [17]	2014	**96.0**	92.5	94.6	**95.0**	10,188	3396	3396	3396
21	SMOTE OUT [34]	2014	93.5	91.2	92.2	92.1	10,188	3396	3396	3396
22	SMOTE Cosine [34]	2014	93.2	89.6	91.2	90.9	10,188	3396	3396	3396
23	Selected SMOTE [34]	2014	94.9	92.7	93.6	93.6	10,188	3396	3396	3396
24	LN SMOTE [35]	2011	94.4	66.7	86.3	93.5	9282	3396	3396	2490
25	MWMOTE [36]	2014	91.5	90.4	91.0	91.0	10,188	3396	3396	3396
26	PDFOS [18]	2014	**95.8**	92.9	94.6	**94.7**	10,188	3396	3396	3396
27	RWO sampling [37]	2014	93.0	88.6	91.0	91.5	10,188	3396	3396	3396
28	NEATER [38]	2014	88.0	75.8	84.8	86.5	8728	3396	3396	1936
29	DEAGO [39]	2015	85.8	85.8	85.8	85.8	10,188	3396	3396	3396
30	MCT [40]	2015	95.4	93.5	94.5	94.5	10,188	3396	3396	3396
31	SMOTE IPF [41]	2015	94.1	92.5	93.2	93.4	10,188	3396	3396	3396
32	OUPS [42]	2016	93.1	91.4	92.0	92.0	9493	3396	3396	2701
33	SMOTE D [43]	2016	81.4	78.7	80.1	80.1	10,189	3398	3396	3395
34	CE SMOTE [44]	2010	94.8	66.7	86.2	90.1	8647	2729	3396	2522
35	Edge Det SMOTE [45]	2010	93.8	92.6	93.2	93.5	10,188	3396	3396	3396
36	ASMOBD [46]	2012	88.2	86.8	87.4	87.3	10,188	3396	3396	3396
37	Assembled SMOTE [47]	2013	93.0	90.9	91.6	91.5	9191	2729	3396	3066
38	SDSMOTE [48]	2014	94.4	92.0	93.4	93.5	10,188	3396	3396	3396
39	G SMOTE [49]	2014	94.4	92.5	93.2	93.2	10,188	3396	3396	3396
40	NT SMOTE [50]	2014	93.7	92.8	93.1	93.1	10,188	3396	3396	3396
41	Lee [51]	2015	93.8	92.9	93.3	93.3	10,188	3396	3396	3396
42	MDO [52]	2016	92.1	90.3	91.3	91.4	10,188	3396	3396	3396
43	Random SMOTE [53]	2011	94.4	92.5	93.3	93.2	10,188	3396	3396	3396
44	VIS RST [54]	2016	66.7	66.6	66.7	66.7	7119	3396	3396	327
45	AND SMOTE [55]	2016	92.0	90.4	91.1	91.0	10,188	3396	3396	3396
46	NRAS [56]	2017	90.2	88.5	89.1	89.0	10,188	3396	3396	3396
47	NDO sampling [57]	2011	95.1	93.6	94.5	94.6	10,189	3397	3396	3396
48	Gaussian SMOTE [58]	2017	92.2	90.3	91.1	91.0	10,188	3396	3396	3396
49	Kmeans SMOTE [59]	2018	92.1	90.8	91.5	91.6	10,188	3396	3396	3396
50	Supervised SMOTE [60]	2014	92.8	91.5	92.1	92.1	10,188	3396	3396	3396
51	SN SMOTE [61]	2012	95.2	92.3	93.7	93.7	10,188	3396	3396	3396
52	CCR [62]	2017	88.9	87.1	88.0	88.2	9191	2729	3396	3066
53	ANS [63]	2017	91.3	88.7	90.0	90.1	9191	2729	3396	3066

The correctly classified points constituting the seabed object were presented in the 10 confusion matrices formed for:

- unbalanced data and data with downsampling (Table 4) for comparison [64],
- four matrices for algorithms with the highest overall classification accuracy (Table 5), and
- four matrices for algorithms with the highest median overall classification accuracy in 11 repetitions (Table 6).

The overall classification accuracy achieved for unbalanced data was 89.6% and for downsampling data was 93.5% [64]. The downsampling method was used, in which each class was given the same number of vectors, similar to in class 3. The data set, divided into three equal classes, contained a total of 219 input vectors (3 × 79). Downsampling contributed to increasing the overall classification accuracy by 3.9%. The correct classification of points in class 3 also increased by 11.3%.

Table 5 and Table 6 present the confusion matrix for the four algorithms with the best object detection results and four algorithms with the best median values. The correct classification of points in class 3 (seabed object) ranged between 89.7% and 93.4% for the best results of imbalanced learning and between 84.9% and 92.5% for median results. In all cases, an increase in the overall classification accuracy and point detection on the seabed objects was achieved. The water surface was classified with an accuracy of 100% in all algorithms. Two algorithms—Safe Level SMOTE and VIS RST—were found to be ineffective and as a result, none of the points on the objects were detected.

The accuracy of oversampling algorithms was assessed using three accuracy evaluation indices: precision, recall, and F1-score.

Precision refers to the proportion of correctly predicted points on the object to all points on the object, i.e.,

(11) $$\mathrm{Precision}=\frac{TP}{TP+FP}\;$$

Recall: refers to the proportion of the correctly predicted points on the object to all points on the object, i.e.,

(12) $$\mathrm{Recall}=\frac{TP}{TP+FN}\;$$

F1-score refers to the harmonic mean of precision and recall, i.e.,

(13) $$F1-\mathrm{score}=\frac{2TP}{2TP+FP+FN}\;$$

where *TP, TN, FP*, and *FN* denote true positive, true negative, false positive, and false negative, respectively.

The indices were computed for the median of results. Recall was found to be high for all four algorithms: 0.92 for LVQ SMOTE, 0.86 for ROSE, 0.85 for borderline-SMOTE2, and 0.84 for PDFOS. The F1-score for class 3 was calculated to be 0.54, 0.66, 0.68, and 0.72, respectively. Among the oversampling algorithms, MDO had the best F1-score of 0.75, which was comparable with that of PDFOS. The overall accuracy of the median results of MDO was 91.4, and the confusion matrix of the median results is presented in Table 7.

## 4. Conclusions

ALB technique follows existing water reservoir measurement patterns. Monitoring the seabed and detection of seabed objects in the coastal zone around ports with heavy vessel traffic help in decreasing the risk of maritime grounding and collision with underwater obstacles, thereby reducing the probability of environmental incidents that can occur due to cargo and fuel leakage or even unexploded ordnance explosion.

This study used a total of 53 oversampling algorithms with imbalanced MLP neural learning for the classification of the ALB data and detection of seabed objects. The results revealed that selected oversampling algorithms classified point clouds better than unbalanced data or data with simple downsampling. The algorithms that produced the best results can be divided into two groups: (1) the algorithms with good recall, which improves the detection of points on objects—LVQ SMOTE and ROSE; and (2) those that improve the general classification with the highest F1-score—MDO and PDFOS. Identifying the oversampling method that gives the best results for object classification and detection is challenging. This is because a good recall is often associated with false classification of points.

As the present study did not cover all the issues related to the subject, future work should focus on using SMOTE methods for improving the detection of underwater objects. Additionally, the possibility of applying SMOTE in deep-sea bottom imaging using MBES would be a topic of interest.

## Figures and Tables

**Table 1:** Description of features used to train the ANN.

U_i_	Description	Formula	
U_1_	Amplitude—the maximal peak of the Gaussian curve and is closely associated with the reflectance intensity [13]		
U_2_	Echo width—(*ω*, full width at half maximum)—the width of a Gaussian curve measured between those points on the *y*-axis which are half the maximal peak, and in the Gaussian function, it is related to standard deviation *σ*	$w=2\sqrt{2\;ln\left(2\right)}\;s$	(1)
U_3_	Return number (*N*)		
U_4_	Number of returns (*N_t_*)		
U_5_	Normalized echo	$N_{z}=\frac{N}{N_{t}}$	(2)
U_6_	Height difference (*dz*)— the vertical distance between the examined point *z_i_* and the lowest *z_min_* in the cylinder	$dz=z_{i}-z_{min}$	(3)
U_7_	Height variance $\left(\sigma^{2}\right)$—a measure of dispersion and is defined as the arithmetic mean of the squares of deviations of individual values $z_{i}$ in the cylinder from the mean value $\bar{z}$	$\sigma^{2}=\frac{1}{n}\overset{n}{\underset{i=1}{\sum }}{\left(z_{i}-\bar{z}\right)}^{2}$	(4)
U_8_	Eigenvalue *λ_1_*		
U_9_	Eigenvalue *λ_2_*		
U_10_	Eigenvalue *λ_3_*		
U_11_	Sphericity—a property that describes the convexity or concavity of the analyzed point relative to points inside the cylinder	$S_{\lambda }=\frac{\lambda_{3}}{\lambda_{1}}$	(5)
U_12_	Planarity—a characteristic that represents the planar aspect of a point arrangement	$P_{\lambda }=\frac{\lambda_{2}-\lambda_{3}}{\lambda_{1}}$	(6)
U_13_	Linearity—a characteristic indicating that the distribution of points is linear (continuous).	$L_{\lambda }=\frac{\lambda_{1}-\lambda_{2}}{\lambda_{1}}$	(7)
U_14_	Eigentropy—defined as entropy computed from eigenvalues	$E_{\lambda }=-\overset{3}{\underset{i=1}{\sum }}\lambda_{i}ln\lambda_{i}$	(8)
U_15_	Omnivariance—a property whose low values are associated with flat terrain or linear structures, while high values are associated with point spatial dispersion [14]	$O_{\lambda }=\sqrt[{3}]{\overset{3}{\underset{i=1}{\prod }}\lambda_{i}}$	(9)

**Table 2:** Description of outputs from the ANN.

U_i_	Description
U_38_	Class 1: “water surface”
U_39_	Class 2: “seabed”
U_40_	Class 3: “seabed object”

**Table 4:** Confusion matrix of unbalanced data and data with downsampling.

Class	Water Surface		Seabed		Seabed Object	
	(Points)	(%)	(Points)	(%)	(Points)	(%)
Unbalance						
Water surface	10,612	100	0	0	0	0
Seabed	0	0	13,057	98.0	261	2.0
Seabed object	0	0	62	29.2	150	**70.8**
Downsampling [64]						
Water surface	10,612	100	0	0	0	0
Seabed	0	0	13,119	98.5	199	1.5
Seabed object	0	0	38	17.9	174	**82.1**

**Table 5:** Confusion matrix of the four algorithms with best object detection.

Class	Water Surface		Seabed		Seabed Object	
	(Points)	(%)	(Points)	(%)	(Points)	(%)
**LVQ SMOTE**						
Water surface	10,612	100	0	0	0	0
Seabed	0	0	12,986	97.5	332	2.5
Seabed object	0	0	14	6.6	198	**93.4**
**ROSE**						
Water surface	10,612	100	0	0	0	0
Seabed	0	0	13,149	98.7	169	1.3
Seabed object	0	0	23	10.8	189	**89.2**
**PDFOS**						
Water surface	10,612	100	0	0	0	0
Seabed	1	0.0	13,143	98.7	174	1.3
Seabed object	0	0	24	11.3	188	**88.7**
**Borderline-SMOTE2**						
Water surface	10,612	100	0	0	0	0
Seabed	6	0.05	13,104	98.4	208	1.6
Seabed object	0	0	26	12.3	186	**87.7**

**Table 6:** Confusion matrix for the algorithms with the highest median.

Class	Water Surface		Seabed		Seabed Object	
	(Points)	(%)	(Points)	(%)	(Points)	(%)
**LVQ SMOTE**						
Water surface	10,612	100	0	0	0	0
Seabed	0	0	13,003	97.6	315	2.4
Seabed object	0	0	16	7.5	196	**92.5**
**ROSE**						
Water surface	10,612	100	0	0	0	0
Seabed	0	0	13,160	98.8	158	1.2
Seabed object	0	0	29	13.7	183	**86.3**
**Borderline-SMOTE2**						
Water surface	10,612	100	0	0	0	0
Seabed	3	0.02	13,175	98.9	140	1.1
Seabed object	0	0	31	14.6	181	**85.4**
**PDFOS**						
Water surface	10,612	100	0	0	0	0
Seabed	1	0.01	13,212	99.2	105	0.8
Seabed object	0	0	32	15.1	180	**84.9**

**Table 7:** Confusion matrix of median results for algorithm MDO.

Class	Water Surface		Seabed		Seabed Object	
	(Points)	(%)	(Points)	(%)	(Points)	(%)
MDO						
Water surface	10,612	100	0	0	0	0
Seabed	0	0	13,266	99.6	52	0.4
Seabed object	0	0	54	25.5	158	74.5

## Data Availability

Not applicable.
